# Monteggia type IV fracture in a child with radial head dislocation irreducible by closed means: a case report

**DOI:** 10.1186/1756-0500-7-539

**Published:** 2014-08-16

**Authors:** Tina Ha, Stephen Grant, James S Huntley

**Affiliations:** Department of Orthopaedics, Royal Hospital for Sick Children, Dalnair Street, Yorkhill, Glasgow, G3 8SJ UK

**Keywords:** Paediatric, Monteggia fracture-dislocation, Irreducible radial head

## Abstract

**Background:**

Fractures of the proximal third of the ulna and radius with associated anterior radial head dislocation are uncommon in children. Early recognition and appropriate management are essential to prevent long-term consequences of loss of forearm rotation, cubitus valgus, elbow instability and chronic pain.

**Case presentation:**

We present the case of a 3-year-old Caucasian boy who attended the emergency department following an un-witnessed fall, resulting in right elbow and forearm pain, swelling and deformity. Clinical and radiological examination revealed a Monteggia type IV fracture-dislocation.

The patient was treated with closed manipulation and percutaneous fixation of both bone forearm fractures with intra-medullary wires. After failed attempts at closed reduction, open reduction of the radial head was required.

The block to reduction was due to a buttonholing of the radial head through the anterior joint capsule, with interposition of the capsule in the radiocapitellar joint. Subsequently, alignment was maintained with fracture healing. Follow-up at five months showed a full range of elbow movement with no adverse symptoms.

**Conclusion:**

Monteggia lesions of the paediatric elbow, albeit uncommon, should be considered in all forearm fractures. Accurate reduction of the radiocapitellar joint is crucial to prevent significant long-term consequences and failed closed reduction requires open reduction. Here we have described the management of a rare type IV lesion in which there was buttonholing of the radial head through the anterior capsule, causing the radiocapitellar dislocation to be irreducible (even after fixation of the radial and ulnar fractures).

## Background

Fracture of the proximal third of the ulna with associated anterior radial head dislocation was first described in 1814 by Giovanni Battista Monteggia, a professor of surgery in Milan. It was not until 1967 that José Luis Bado developed a classification scheme according to the anatomical variations of the fracture-dislocation [1].

These lesions are uncommon in the paediatric setting, especially the type 4 (i.e., with radial fracture also). Early recognition and management are required to prevent the long-term consequences of persistent radio-ulnar dislocation.

## Case presentation

A 3-year-old Caucasian boy presented with his mother to the emergency department complaining of pain and deformity in his right arm following an unwitnessed fall whilst playing in the garden.

**Figure 1:**
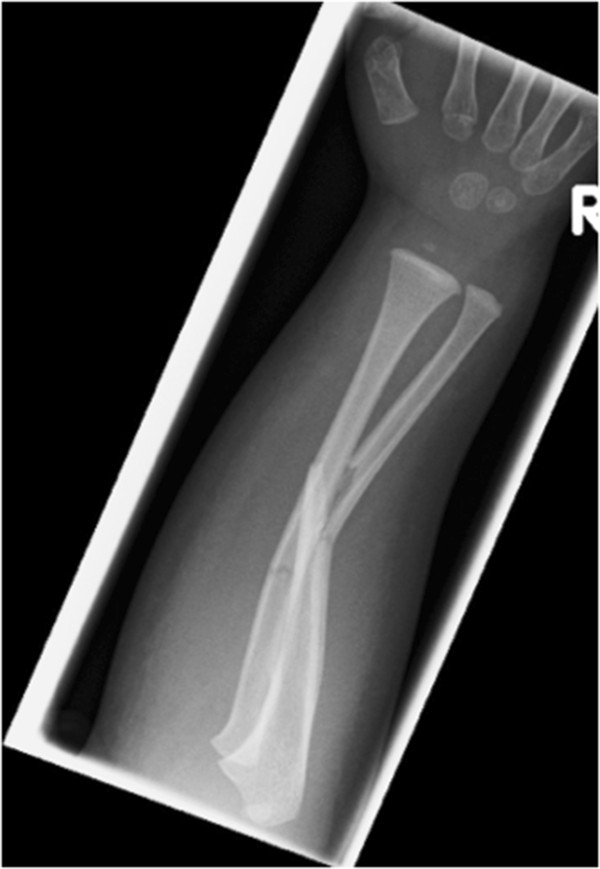
**Pre-operative radiographs demonstrating displaced both bone forearm fractures.**

**Figure 2:**
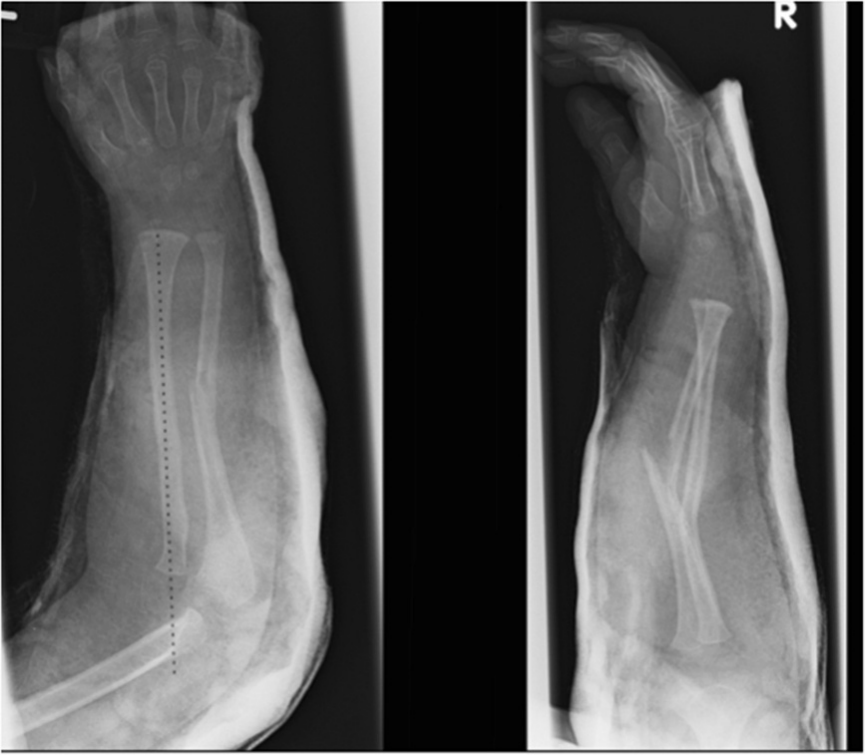
**Subsequent further imaging in plaster demonstrating an anterior dislocation of the radiocapitellar joint.**

**Figure 3:**
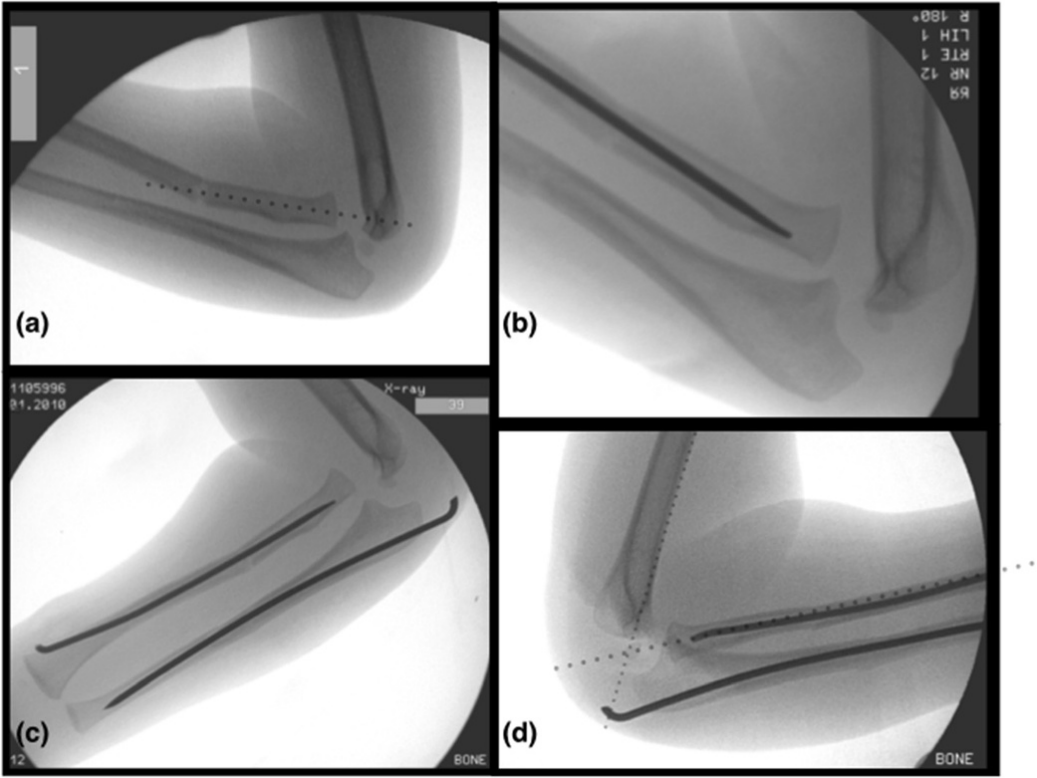
**Intra-operative screening images demonstrating the sequence of steps resulting in successful reduction of the fractures and radiocapitellar joint.** **(a)** Closed reduction of ulna did not relocate radial head; **(b)** Proceeded onto reduction of radius with 2 mm titanium elastic nail but radiocapitellar joint remained dislocated; **(c)** Anatomic reduction of ulna with percutaneous 2 mm stainless steel wire also did not result in reduction; **(d)** After open reduction via a postero-lateral approach resulting in successful reduction.

**Figure 4:**
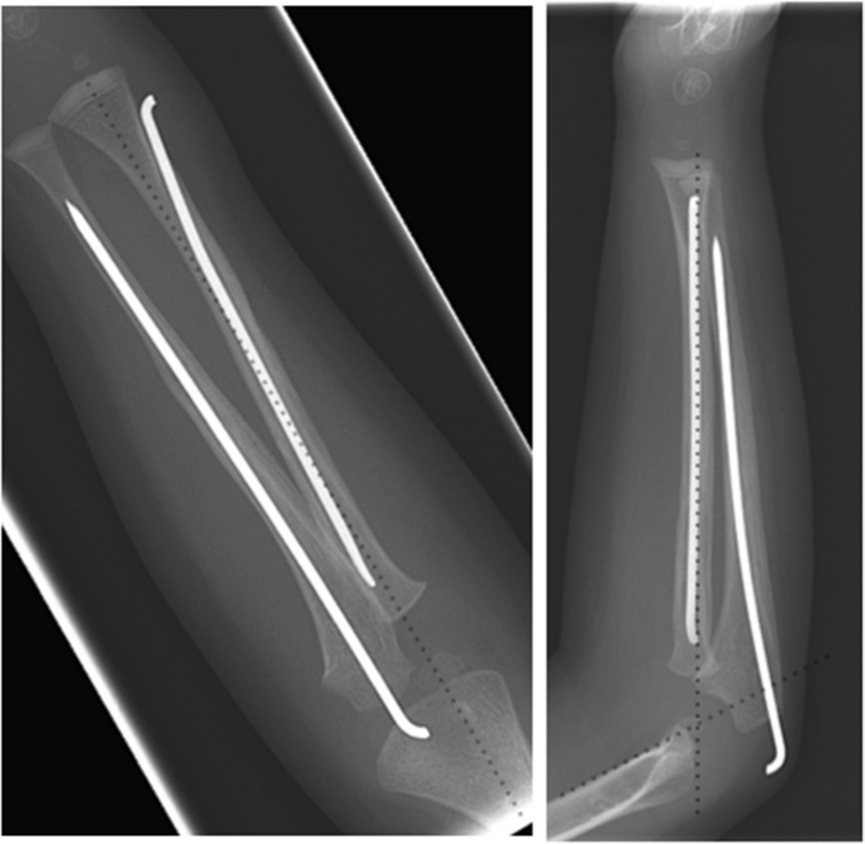
**8 weeks post-operative radiographs demonstrating union of both radius and ulnar shaft fractures with successful maintenance of radiocapitellar joint reduction.**

There was deformity of the right forearm with a small abrasion over the proximal ulna. His peripheral pulses and capillary refill time were normal. However, the patient was upset and uncooperative and therefore the neurological status of the affected limb was impossible to assess comprehensively. There was no history of previous fractures, dislocations or any medical conditions.Initial radiographs showed diaphyseal fractures of both radius and ulna (Figure 1). After immobilisation, further radiographs of the elbow revealed a Bado type IV Monteggia lesion – fractures of both radius and ulna with an associated anterior dislocation of the radial head (Figure 2).The following day, the injury was treated with manipulation of the forearm fractures under general anaesthesia (Figure 3a). Closed reduction of the forearm fractures was unsuccessful at reducing the radiocapitellar joint (RCJ) dislocation so a 2 cm radial incision was made over the distal radius and a 2 mm titanium elastic nail (TEN) was inserted under X-ray image intensifier (XRII) guidance across the radial fracture site. Despite successful reduction of the radial fracture, the RCJ remained dislocated (Figure 3b). A 2 mm Kirschner wire (K-wire) was then inserted across the ulnar fracture site via the olecranon apophysis (Figure 3c), but again the RCJ was not reducible. A decision was made to carry out an open reduction of the RCJ. A posterolateral approach revealed a radial head which had buttonholed through the capsule, associated with a ruptured annular ligament. The capsule was also found interposed within the joint and these findings explained the failed attempts at closed reduction.The interposed tissue was freed from the joint, the radial head reduced and the position confirmed using XRII. Reducing the radial head in this case required placement of two McDonald retractors along the radial neck, through the buttonhole in the capsule and then at right angles to each other (one around the radial head in the axial plane and one over the radial head proximally in the sagittal plane). Using these two levers, the radial head was brought back through the buttonhole, clearing the obstructing capsular mass from the articulation. The RCJ reduced and was then stable throughout a full range of movement. The annular ligament was reconstructed and the forearm immobilised in an above elbow cast in flexion and mid-supination (Figure 3d).Follow-up at one week showed no change in alignment of the RCJ and no neurovascular abnormalities. In particular, there was normal posterior interosseous nerve (PIN) function with extension of all fingers at the metacarpo-phalangeal joints. He remained in cast until his six week follow-up where radiographs showed normal radiocapitellar alignment and ongoing fracture healing, at which point he was fitted with a below elbow cast. Follow-up at eight weeks showed satisfactory radiographic union and maintenance of reduction and his cast was removed (Figure 4). He had full range of movement at his right elbow and had wire removal from both radius and ulna under general anaesthesia 12 weeks following the injury. Follow-up at five months demonstrated full range of elbow movement and no adverse symptoms.

## Discussion

We have described an unusual case of a Monteggia type IV lesion (itself a rarity) with radial head buttonholing through the anterior joint capsule. Early recognition and imaging of the elbow associated with forearm fractures is crucial, as persistent radial head dislocation can result in loss of forearm rotation, cubitus valgus, elbow instability and chronic pain [2–4]. This is especially important in younger and uncooperative children in whom assessment may be difficult in the acute setting.

The initial emergency department radiographs did not allow visualisation of the elbow joint and the radial head dislocation could easily have been missed. For this reason, it is imperative that in diaphyseal forearm fractures, the elbow is also examined clinically and radiographically.

All patients should also be examined for associated nerve injury, most commonly the radial nerve involving the PIN. This results in weakness of the wrist and finger extensors, as well as the supinator but no sensory deficit. Fortunately, most nerve palsies associated with Monteggia lesions resolve spontaneously with reduction of the radiocapitellar joint [5]. Sometimes, especially in the very young, comprehensive neurological evaluation is impossible. In such a case, it must be documented as such.

Bado classified Monteggia fractures into four groups, reporting that type I was the most common, with type IV Monteggia lesions being a relatively rare injury [6]. Closed reduction of the RCJ dislocation is generally successful once the diaphyseal fractures have been reduced, especially in greenstick fractures, but if this is not achieved, open reduction is required.

In this case, the logical sequence of events that took place included:

1. Closed reduction with longitudinal traction and supination in an attempt to convert the type IV lesion into a type I lesion.
2. Once this had failed, closed anatomical reduction and fixation of the radial and ulnar fractures was carried out but again failed to reduce the radial head dislocation, indicating interposition of soft tissue.
3. Open exploration of the RCJ and reduction of the radial head, which was successful. Paradoxically, fixation of the radial fracture made the open reduction of the RCJ dislocation more difficult as the fixation held the radius out to correct length. It therefore took greater effort to lever the radial head back through the ruptured anterior joint capsule. Nevertheless, we would still proceed in the aforementioned steps above.

Structures obstructing anatomical reduction have been reported, including the capsule, annular ligament and biceps tendon [7–9]. What was unusual about this case, in addition to it being a type IV lesion, was the buttonholing of the radial head through the anterior capsule.

It is imperative that the operating surgeon is aware of the possible pitfalls that this fracture pattern can present in the paediatric population. Knowledge of the sequence of intra-operative steps needed to be followed to successfully reduce the RCJ is important and recognition of the situations that require open reduction.

## Conclusion

We have discussed a case of irreducible radial head dislocation following a Monteggia type IV lesion that required open reduction. These are uncommon but there are several learning points from this case:

1. Monteggia type lesions of the paediatric elbow should be considered in forearm fractures and initial assessment should include examination of the elbow and imaging where appropriate.
2. Irreducibility of the dislocation should alert the surgeon to the presence of interposed soft tissue and prompt the need for open reduction.

## Consent

Written informed consent was obtained from the patient’s legal guardian for publication of this case report and accompanying images. A copy of the written consent is available for review by the Editor-in-Chief of this journal.

## Abbreviations

K-wire: Kirschner wire
MCPJ: Metacarpophalangeal joints
PIN: Posterior interosseous nerve
RCJ: Radiocapitellar joint
TEN: Titanium elastic nail
XRII: X-ray image intensifier.
